# Disparities in Janus kinase inhibitor access for alopecia areata: a retrospective analysis

**DOI:** 10.1097/JW9.0000000000000155

**Published:** 2024-06-10

**Authors:** Elizabeth J. Klein, Dolly Taiwò, Efe Kakpovbia, Melissa Laughter, Ambika Nohria, Kristen I. Lo Sicco

**Affiliations:** a The Ronald O. Perelman Department of Dermatology, NYU Grossman School of Medicine, New York, New York

**Keywords:** alopecia areata, disparities, insurance approval, JAK-STAT inhibitors

**What is known about this subject in regard to women and their families?**

- Alopecia areata (AA) affects a disproportionate number of women compared to men worldwide.
- The impacts of AA on women cannot be understated and include physical, psychosocial, and financial impacts.
- As women are often the primary caregivers in the household, pediatric AA also poses a significant challenge for women, particularly regarding providing financial and emotional support for children.

**What is new from this article as messages for women and their families?**

- This article highlights the significant challenges that AA patients face when trying to obtain treatment.
- The majority of patients included in this study were female (59%).
- Our findings revealed that patients were frequently denied initial insurance approval or required multiple rounds of appeals to obtain coverage.
- This contributes to the already high levels of emotional and financial stress associated with AA.

## Introduction

Janus kinase inhibitor (JAKi) use has grown rapidly in dermatology.^1^ JAKi is currently the only Food and Drug Administration-approved therapy for severe alopecia areata (AA). However, barriers including insurance denial and prescription costs may preclude access to JAKi and threaten to exacerbate pre-existing racial and socioeconomic disparities in access to dermatologic care.^2,3^ This study aims to identify and characterize factors that limit access to JAKi among AA patients.

## Methods

We performed a retrospective chart review of 115 patients seen at New York University Langone Health from January 2013 to May 2023. We extracted patient demographics, specialty of prescriber, indication for prescription, insurance status, prior authorization (PA) outcomes, and time from initial prescription to medication initiation. Statistical analyses were performed with SPSS 28.0.0.0 (IBM headquartered in Armonk, New York).

## Results

**Fig. 1.**
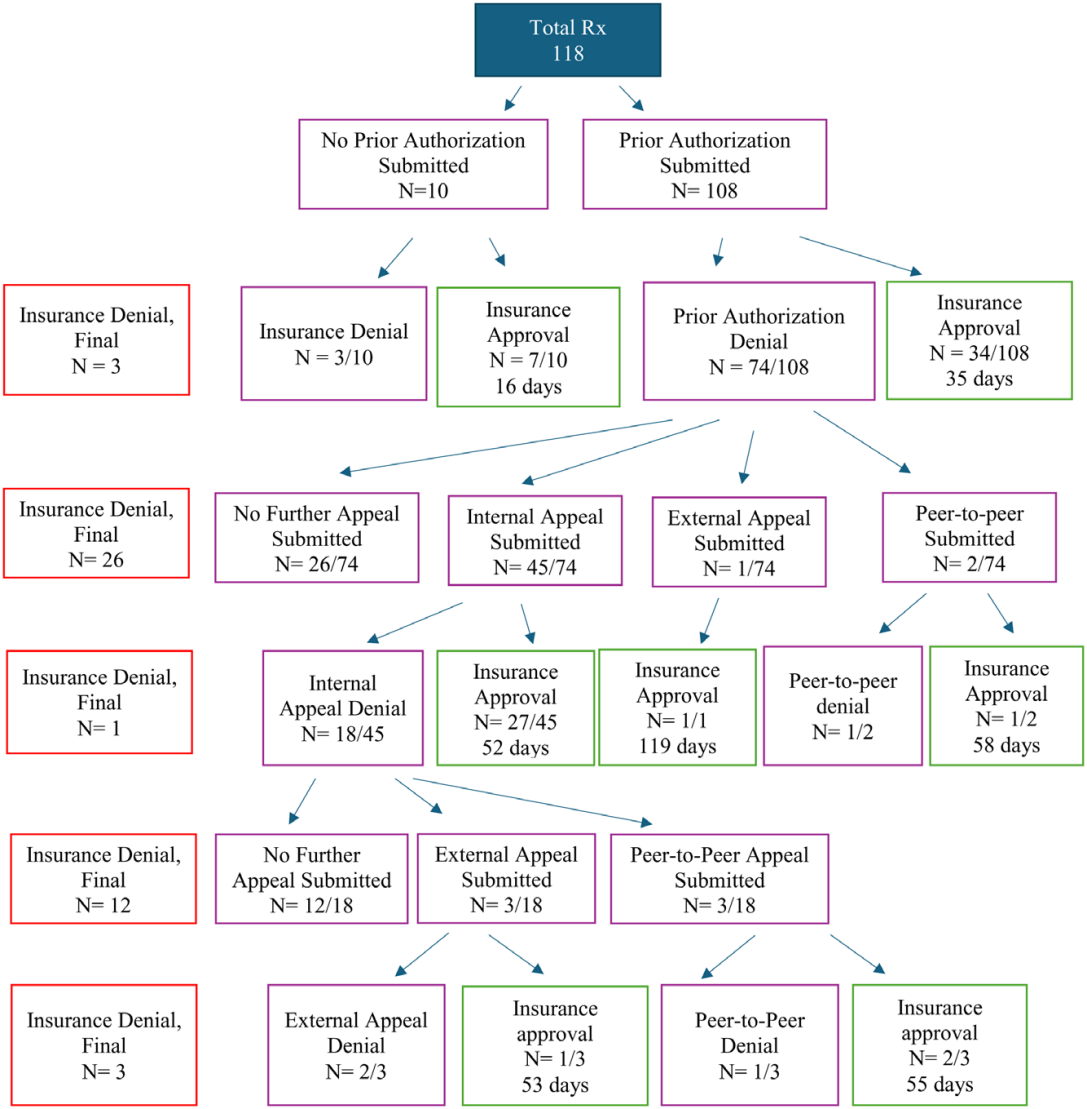
Pathway and mean days to JAK-STAT insurance approval.

A total of 100 patients receiving 118 unique JAKi prescriptions met the inclusion criteria (Table 1). AA was the primary indication for 92% of prescriptions, and the prescribing physician was a dermatologist for 93%. Of the 118 prescriptions submitted, 62% ultimately received insurance approval (Fig. 1). Of those approved, 91% required a PA and 44% required 2 or more levels of appeal. A significantly greater proportion of patients with public health insurance were ultimately denied coverage compared with patients with private health insurance (67% vs 34%, *P* =.028).

**Table 1:** Patient characteristics and prescribing data

Patient characteristics	Overall, *N* = 100, Rx = 118
Sex	*N* = 100
Female, *n* (%)	59 (59%)
Male, *n* (%)	41 (41%)
Age (yrs, ±SD)	33 (7, 76)
Race/ethnicity	*N* = 100
Non-Hispanic White, *n* (%)	44 (44%)
Non-Hispanic Black, *n* (%)	7 (7%)
Hispanic, Latinx, *n* (%)	9 (9%)
Non-Hispanic Asian, *n* (%)	4 (4%)
Other, *n* (%)	3 (3%)
Unknown race, *n* (%)	33 (33%)
Alopecia areata subtype	*N* = 100
Patch type	54 (54%)
Ophiasis	17 (17%)
Totalis	10 (10%)
Universalis	7 (7%)
Unknown	12 (12%)
Insurance type	*N* = 100
Private insurer	87 (87%)
Medicare	2 (2%)
Medicaid or state public health insurer	10 (10%)
International insurance	1 (1%)
Indication for JAK-STAT prescription	Rx = 118
Alopecia areata	109 (92%)
Atopic dermatitis	4 (3%)
Psoriatic arthritis	2 (2%)
Vitiligo	2 (2%)
Rheumatoid arthritis	1 (1%)
Dermatomyositis	1 (1%)
SLE	1 (1%)
Ulcerative colitis	1 (1%)
JAK-STAT inhibitor prescribed	Rx = 118
Tofacitinib, oral	60 (51%)
Ruxolitinib, topical	27 (23%)
Baricitinib, oral	28 (24%)
Tofacitinib, topical	2 (2%)
Upadacitinib, oral	1 (1%)
Specialty of prescribing physician	Rx = 118
Dermatologist	110 (93%)
Rheumatologist	7 (6%)
Gastroenterologist	1 (1%)
JAK-STAT inhibitor accessed without insurance	Rx = 43/118
Tofacitinib, oral	25/60
Free samples	7/60
Out of pocket	5/60
Assistance program	13/60
Ruxolitinib, topical	7/27
Free samples	2/27
Out of pocket	1/27
Assistance program	4/27
Baricitinib, oral	11/28
Free samples	5/28
Out of pocket	2/28
Assistance program	4/28
Tofacitinib, topical	0/6
Free samples	0/2
Out of pocket	0/2
Assistance program	0/2
Upadacitinib, oral	0/3
Free samples	0/1
Out of pocket	0/1
Assistance program	0/1

Rx, prescriptions; SD, standard deviation; SLE, systemic lupus erythematosus.

Among prescriptions initially or ultimately denied, there were 43 instances of patients obtaining the medication through provision of free samples, a financial assistance program, or out-of-pocket payments (Table 1). Patients who obtained JAKi through these pathways had significantly longer wait times for treatment initiation compared with patients who received insurance approval (162 days vs 42 days, *P* =.002). Male patients also had significantly longer waiting periods (125 days vs 45 days, *P* =.028). Commercial insurance holders waited longer than public insurance holders, although this difference was not statistically significant. We found no significant associations between insurance approval or time to medication initiation and patient race, type of JAKi prescribed, specialty of prescribing physician, medication indication, or AA subtype, though most of our cohort had commercial insurance and were Caucasian. Further studies with larger sample sizes may elucidate the interplay between these factors and the insurance approval process.

## Conclusion

This work highlights barriers to initiating JAKi therapy for AA patients. Patients were frequently denied initial insurance approval or required multiple rounds of appeals. These obstacles resulted in significant delays in starting treatment for many. Patients with public health insurance were significantly more likely to be denied insurance approval for their medication, suggesting that demographic factors may impact access.^4^ Beyond patient barriers, obtaining JAKi also imposes a significant administrative burden. In our practice, on average, completing a PA takes at least 30 minutes, internal and external appeals at least 1 hour, and peer-to-peer conversations at least 1 hour. Cumulatively, this represents a significant administrative burden, often requiring additional dedicated staff members, at our practice approximately 4, to complete. Identifying these patient and administrative barriers is critical for informing policy decisions to improve equitable access to dermatologic care. Study limitations include retrospective design and limited sample size.

## Conflicts of interest

None.

## Funding

None.

## Study approval

The authors confirm that any aspect of the work covered in this manuscript that has involved human patients has been conducted with the ethical approval of all relevant bodies.

## Author contributions

EJK, DT, EK, ML, and AN contributed to the data collection, analysis, and drafting of this manuscript. KILS contributed to the project development and review of manuscript.
